# Identification and characterization of *SEC24D* as a susceptibility gene for hepatitis B virus infection

**DOI:** 10.1038/s41598-019-49777-8

**Published:** 2019-09-17

**Authors:** Xianzhong Jiang, Bin Zhang, Junsheng Zhao, Yi Xu, Haijun Han, Kunkai Su, Jingjing Tao, Rongli Fan, Xinyi Zhao, Lanjuan Li, Ming D. Li

**Affiliations:** 1 State Key Laboratory for Diagnosis and Treatment of Infectious Diseases, Collaborative Innovation Center for Diagnosis and Treatment of Infectious Diseases, The First Affiliated Hospital, Zhejiang University School of Medicine, Hangzhou, China; 2 Research Center for Air Pollution and Health, Zhejiang University, Hangzhou, China

**Keywords:** Genetic markers, Hepatitis B

## Abstract

Single nucleotide polymorphisms (SNPs) and genes associated with susceptibility to hepatitis B virus (HBV) infection that have been identified by genome-wide association studies explain only a limited portion of the known heritability, indicating more genetic variants remain to be discovered. In this study, we adopted a new research strategy to identify more susceptibility genes and variants for HBV infection. We first performed genetic association analysis of 300 sib-pairs and 3,087 case-control samples, which revealed that 36 SNPs located in 31 genes showed nominal associations with HBV infection in both samples. Of these genes, we selected *SEC24D* for further molecular analysis according to the following two main lines of evidence. First, a time course analysis of the expression profiles from HBV-infected primary human hepatocytes (PHH) demonstrated that *SEC24D* expression increased markedly as time passed after HBV infection (*P* = 4.0 × 10^−4^). Second, SNP rs76459466 in *SEC24D* was adversely associated with HBV risk (OR_meta_ = 0.82; *P*_meta_ = 0.002), which again indicated that *SEC24D* represents a novel susceptibility gene for HBV infection. Moreover, *SEC24D* appeared to be protective against HBV infection *in vitro*. Consistently, we found that *SEC24D* expression was significantly enhanced in non-infected liver tissues (*P* = 0.002). We conclude that *SEC24D* is a novel candidate gene linked to susceptibility to HBV infection.

## Introduction

Hepatitis B virus (HBV) infection is a serious global health issue and shows marked geographic diversity. Thus, in the United States and northern European countries, the prevalence of chronic HBV infection (CHBVI) is estimated to be <0.5%, but this figure is as high as 10–12% in China and South Korea^1,2^. Despite the availability of a potent HBV vaccine and effective antiviral drugs for two decades, hepatitis B maintains at high prevalence worldwide, with more than 240 million people infected^3^. Chronic hepatitis B can cause liver cirrhosis and hepatocellular carcinoma and is responsible for more than 0.5–1.0 million deaths per year^4^.

Chronic HBV infection (CHBVI) and viral clearance are influenced by multiple genetic and environmental factors, including viral and host factors^5–7^. Twin and segregation studies indicate that host genetic components strongly influence the outcome of HBV infection^8^. Recently, genome-wide association study (GWAS) has been used to identify genetic variants for numerous complex human diseases such as HBV infection and clearance. Several loci, located in human leukocyte antigen-C (*HLA-C*)^9^, *HLA-DP*^10^, *HLA-DQ*^11^, *HLA-DOA*, complement factor B (*CFB*), *NOTCH4*^12^, euchromatic histone lysine methyltransferase 2 (*EHMT2*), transcription factor 19 (*TCF19*)^13^, and two non-HLA loci, *CD40*^12^ and ubiquitin conjugating enzyme E2 L3 (*UBE2L3*)^9^, have been reported to be significantly associated with HBV-related diseases. However, as observed in many human disorders, these single nucleotide polymorphisms (SNPs) account for only a small proportion of the apparent genetic variance, implying many susceptibility loci remain to be identified for HBV-related diseases^14^.

To further elucidate disease-predisposing genes for HBV infection, we employed an integrative functional genomics strategy, which can be summarized briefly as follows. We first conducted a variant discovery in 300 sib-pairs, followed by replication of top candidate SNPs in 3,087 case-control samples. The *SEC24D* gene was then selected for analysis based on the expression analysis and association study. Through a series of *in vitro* experiments, we found *SEC24D* to be an antiviral gene for HBV infection.

## Methods

### Subjects

The three hundred sib-pairs used for the discovery stage were recruited between 2010 and 2012 at the First Affiliated Hospital of Zhejiang University School of Medicine and other neighbor medical hospitals or centers. Among these siblings, 300 CHBVI subjects were defined as seropositive for either hepatitis B surface antigen (HBsAg) or HBV-DNA, and the corresponding sib-controls were negative for both. More detailed descriptions of the demographic and phenotypic characteristics of these subjects are shown in Table 1 and Supplementary Fig. 1A.

**Table 1:** Demographic characteristics of samples used in this study.

Characteristic	Family sample		Population sample	
	CHBVI	Sib-Controls	CHBVI	Unrelated Controls
Sample size	300	300	1648	1439
Age (years)	39.5 ± 11.3	39.3 ± 11.7	40.7 ± 12.0	38.2 ± 12.1
Female (%)	110 (36.7%)	169 (56.3%)	428 (26.0%)	535 (37.2%)
HBV-DNA (×10^8^ copies/mL)	6.6 ± 46.1	—	2.0 ± 8.4	—
HBsAg (×10^3^ IU/mL)	1.2 ± 1.8	—	2.8 ± 3.4	—
Albumin (g/L)	44.0 ± 7.2	47.7 ± 3.7	52.6 ± 168.1	47.9 ± 6.6
ALT (U/L)	80.9 ± 127.2	22.0 ± 15.2	66.3 ± 127.2	25.3 ± 26.4
TBIL (μmol/L)	44.3 ± 97.3	11.4 ± 5.7	35.7 ± 242.1	13.3 ± 16.7

Notes: Continuous variables are shown as mean ± standard deviation.

*ALT* = alanine aminotransferase; *CHBVI* = chronic hepatitis B virus infection; *TBIL* = total bilirubin.

The replication sample was recruited from the same medical facilities and included 1,648 CHBVI participates and 1,439 unrelated controls (Table 1 and Supplementary Fig. 1B). A detailed description of this independent replication sample has been provided in previous publications from our group^15,16^.

All the participants were of Chinese Han ethnicity. Informed written consent was obtained from every participant, and the demographic and clinical data were collected by structured questionnaires. This project was approved by the Ethical Committee of the First Affiliated Hospital of Zhejiang University School of Medicine.

### Whole exome-sequencing analysis in the discovery sample

Genomic DNA was extracted from 3 ml of peripheral blood from each subject using the Qiagen DNA purification kit. Libraries were prepared according to the operational manual provided by the manufacturer, and the enriched coding exons were captured using a TruSeq Exome Enrichment Kit (Illumina, San Diego, USA) and sequenced by the Illumina HiSeq2000 system. Paired-end sequencing was carried out for 100 bases from each end of about 200-bp insert fragment libraries using standard Illumina protocols, and sequencing reads were aligned to hg19 from UCSC Genome Browser (http://genome.ucsc.edu/) using the Burrows-Wheeler Aligner (BWA) with default parameters^17^. After removing PCR duplicates by Picard tools (http://broadinstitute.github.io/picard/), the median sequencing depth of all samples was 56 × (see Supplementary Fig. 2). Of the targeted exon regions, 90.73% were covered at an average of ≥10× with genotype quality scores of ≥30. Single nucleotide variations (SNVs) were identified by the Genome Analysis Toolkit (GATK)^18,19^. The statistics of each variant, including allele balance, depth of coverage, strand balance, and multiple quality metrics, were annotated using the GATK Variant Annotator^18,19^. These statistics were then used in an adaptive error model to estimate the probability that each SNV is a true one using the GATK Variant Quality Score Realibrator (VQSR)^18,19^. Functional annotation of variants was performed using the ANNOVAR^20^, and the annotation database was downloaded from the UCSC Genome Browser.

Stringent quality control steps were performed to ensure robust association analysis. Single nucleotide polymorphisms were excluded from further analysis if they had a minor allele frequency (MAF) of <0.01 and a *P* value of <1 × 10^−6^ for Hardy-Weinberg equilibrium (HWE). After this appropriate quality control, a total of 98,357 SNPs remained for further analysis.

### Genotyping in the replication stage

All of the 4,000 SNPs selected at the discovery stage were genotyped using the Illumina iSelect custom genotyping array according to the Illumina Infinium HD Assay Ultra Manual. Among them, 121 SNPs failed to be designed in the custom array. In addition, 291 ancestry informative markers (AIMs) from different chromosomes were included in the iSelect array and used to assess population admixture for the replication samples. An SNP was excluded if it had: (1) a call rate of <0.95; (2) an MAF of <0.05; and (3) a *P* value of <1 × 10^−6^ with the HWE test in the replication samples. Those SNPs located on sex chromosomes also were excluded. Any sample was removed if it had a call rate of <0.95. After these quality control steps, 2,925 SNPs on autosomal chromosomes from 3,064 samples remained for further analysis.

### Gene expression analysis

Gene expression profiles of three independent datasets were downloaded from Gene Expression Omnibus (GEO). Dataset 1 (Accession Number GSE72068) was used to perform time course analysis, which consists of determining 20 mRNA expression profiles in primary human hepatocytes (PHH) obtained at different times after HBV infection using an Illumina HumanHT-12 V4.0 expression beadchip. Dataset 2 (Accession Number GSE36250) was used to measure the differential expression of candidate genes of interest, which consists of examining 123 mRNA expression profiles of liver samples by the NimbleGen Custom Gene Expression HX3 Microarray. Dataset 3 (Accession Number GSE22058) was used for pathway enrichment analysis, which consists of study of expression data from 96 liver specimens from patients with HBV-related HCC employing the Rosetta/Merck Human RSTA Custom Affymetrix 1.0 microarray. The specimens were divided into two groups on the basis of mean *SEC24D* expression. The up quartile was defined as the *SEC24D* high-expression group and the down quartile as the low-expression group. The genes with a false-discovery rate Q value < 0.001 and |fold change| >1.5 were considered highly differentially expressed. Pathway enrichment analysis was carried out with the DAVID tool (v. 6.8)^21^.

### Cells culture and transfection

The human hepatoma cell lines HepG2 and HepG2.2.15 were purchased from the China Center for Type Culture Collection (CCTCC). All cells were cultured in Dulbecco Modified Eagle Medium (HyClone, Logan, UT USA) containing 10% fetal bovine serum (GIBCO, Waltham, MA USA), and penicillin G 100 U/ml and streptomycin 100 μg/ml (GIBCO) at 37 °C in a humidified incubator with 5% CO_2_. The HepG2.2.15 cells were supplemented with G418 400 μg/ml (GIBCO) to maintain the stably transfected dimeric HBV-DNA. The HBV-producing plasmid pGEM-4Z-HBV1.3, which contains 1.3 U of the HBV genome (subtype ayw)^22^, was a gift from Dr. Shick Ryu Wang (Addgene plasmid # 65459). The *SEC24D* cDNA was cloned into the *Sac*II and *Eco*RI sites of the expression vector pEGFP-C3 (Clontech, Palo Alto, CA USA). The recombinant plasmid was sequenced to confirm the accuracy by Sangon Biotech (Shanghai, China). For inhibition of gene expression, the cells were transfected with siRNA duplexes, which were synthesized by RiboBio Inc. (Guangzhou, China). All the transfection reactions were established using the Lipofectamine 3000 Transfection Reagent (Invitrogen, Carlsbad, CA USA) according to the manufacturer’s instruction.

### Western blotting analysis

After 48 h of transfection, cell lysates were collected using a RIPA lysis buffer with protease inhibitors (Tiangen, Beijing, China). The lysates, containing 12 μg of protein, were separated on SDS-PAGE and transferred to PVDF membranes (Millipore, Bedford, USA). The membrane was probed with a designated primary antibody (anti-*SEC24D* and anti-beta actin; Abcam, Cambridge, UK) overnight at 4 °C and further incubated with the corresponding horseradish peroxidase-conjugated secondary antibody (Bioker, Hangzhou, China) for 1 h at room temperature. The immunoreactive bands were labeled with Clarity Western ECL Substrate (Bio-Rad, Richmond, USA). Beta-actin was used as a protein loading control. The signal intensity was quantified by ImageJ software (National Institutes of Health, Bethesda, MD USA).

### Detection of HBV-DNA, HBsAg, and HBeAg

After 48 h of transfection, cell supernatant liquid was collected by centrifugation at 3,000 rpm for 10 min at 4 °C. The HBV DNA load was measured by quantitative real-time PCR using the Fluorescence Quantitative PCR Detection Kit for HBV-DNA (Acon, Hangzhou, China). The HBsAg and HBeAg concentrations were quantified by the chemiluminescent microparticle immunoassay using an ARCHITECT Reagent Kit (Abbott, Chicago, IL USA). All the assays were performed at least three times following the manufacturer’s instructions.

### Data analysis

We carried out a liberalization of the sibling transmission/disequilibrium test (sTDT)^23^ for 300 sib-pairs under an additive genetic model adjusted for age, sex, and the first five principal components (PCs). In the replication stage, association of SNPs with HBV infection was performed under an additive genetic model using PLINK (v. 1.07)^24^ with age, sex, and the first five PCs as covariates. The population admixture of samples was assessed by PC analysis (PCA) as implemented in EIGENSTRAT^25^. Meta-analysis of the data generated from family and case-control samples was carried out to assess the pooled genetic effects using the Mantel-Haenszel method^26^. Heterogeneity was examined with Cochran’s *Q* test^27^. When the *P* value of the *Q* test was < 0.1, we considered there to be strong evidence for heterogeneity between samples. Time course analysis was performed using BRB-ArrayTools software^28^. For signal intensity of WB analysis, *SEC24D* expression, HBV-DNA load, and HBsAg and HBeAg concentrations, significant difference was determined by the two-tailed Student’s *t*-test. A *P* value < 0.05 was considered statistically significant.

## Results

### Association study and expression analysis identified *SEC24D* as a candidate gene for susceptibility to HBV infection

**Figure 1:**
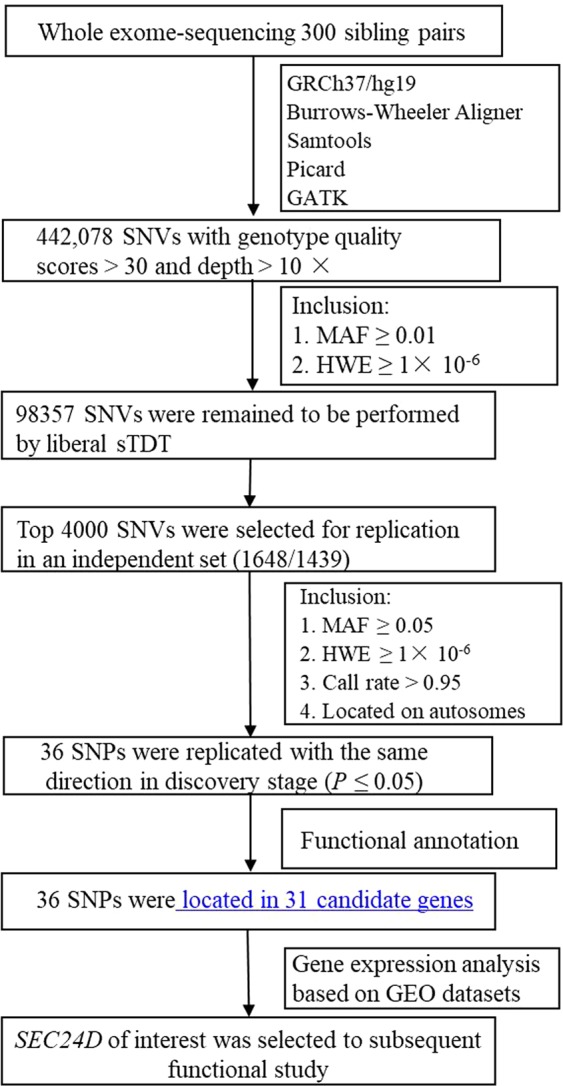
Workflow of the integrative functional genomics methodology to identify the susceptibility gene for HBV infection. Abbreviations: SNV = single nucleotide variation; SNP = single nucleotide polymorphism; MAF = minor allele frequency; HWE = Hardy-Weinberg equilibrium; sTDT = sibling transmission/disequilibrium test.

As shown in Fig. 1 and Table 2, we carried out association analyses for both the family and the case-control samples. First, 442,078 SNVs were identified by WES analysis. Second, we performed sTDT for the 98,357 SNPs remaining after quality controls. Third, the top 4,000 SNPs were selected for replication in 1648 CHBVIs and 1439 unrelated controls, which revealed that 36 SNPs across 31 genes were nominally associated with HBV infection in both samples (all *P* values < 0.05).

**Table 2:** Summary of 36 SNPs in 31 genes associated with HBV infection based on two datasets.

Gene	SNP ID	Chr.: position	Minor allele	MAF		OR (95% CI)	*P* value	MAF		OR (95% CI)	*P* value
				CHBVI	Sib Control			CHBVI	Unrelated Control		
*SEC24D*	rs76459466	chr4: 119652606	T	0.157	0.197	0.64 (0.46–0.88)	0.006	0.162	0.160	0.86 (0.75–0.99)	0.034
AKR1C3	rs3209896	chr10: 5149703	A	0.257	0.193	1.45 (1.10–1.92)	0.009	0.256	0.226	1.18 (1.05–1.33)	0.006
CPLX4	rs75707948	chr18: 56963697	A	0.273	0.217	1.47 (1.10–1.96)	0.009	0.267	0.236	1.20 (1.07–1.36)	0.002
TTLL3	rs1057278	chr3: 9876919	A	0.032	0.063	0.47 (0.26–0.85)	0.012	0.041	0.060	0.78 (0.63–0.98)	0.029
NEB	rs61730780	chr2: 152506811	T	0.053	0.023	2.34 (1.20–4.57)	0.013	0.065	0.051	1.30 (1.04–1.63)	0.023
CPLX4	rs9966780	chr18: 56963111	A	0.258	0.210	1.42 (1.07–1.90)	0.017	0.268	0.236	1.21 (1.07–1.36)	0.002
AIPL1	rs8075035	chr17: 6331803	T	0.402	0.453	0.74 (0.58–0.95)	0.018	0.430	0.446	0.90 (0.81–1.00)	0.042
SIRT4	rs61748115	chr12: 120741382	A	0.113	0.077	1.63 (1.08–2.45)	0.019	0.101	0.098	1.24 (1.04–1.47)	0.015
CALM1	rs3179089	chr14:90873412	G	0.167	0.217	0.70 (0.52–0.94)	0.019	0.194	0.218	0.85 (0.75–0.96)	0.010
ELOVL2	rs17606561	chr6: 10982359	A	0.197	0.238	0.71 (0.54–0.95)	0.021	0.224	0.242	0.89 (0.79–1.00)	0.044
OR4C15	rs12225462	chr11: 55322638	T	0.235	0.300	0.73 (0.56–0.95)	0.021	0.246	0.267	0.89 (0.79–1.00)	0.043
CD2AP	rs1043276	chr6: 47594002	C	0.313	0.268	1.36 (1.05–1.77)	0.022	0.317	0.285	1.19 (1.06–1.33)	0.003
TTLL3	rs1057281	chr3: 9876987	A	0.035	0.065	0.52 (0.29–0.91)	0.023	0.041	0.060	0.79 (0.63–0.98)	0.033
ASB6	rs11540632	chr9: 132397252	A	0.308	0.263	1.36 (1.04–1.78)	0.023	0.300	0.272	1.13 (1.01–1.26)	0.040
NEF	rs142805831	chr21: 48018929	A	0.057	0.032	2.01 (1.09–3.68)	0.025	0.069	0.056	1.27 (1.03–1.57)	0.026
OR5D14	rs76383258	chr11: 55563336	T	0.245	0.302	0.74 (0.57–0.96)	0.026	0.245	0.269	0.88 (0.78–0.98)	0.024
SIRT4	rs16950058	chr12: 120750517	G	0.117	0.082	1.59 (1.06–2.41)	0.027	0.102	0.099	1.24 (1.05–1.48)	0.013
TADA3	rs293778	chr3: 9833986	T	0.028	0.055	0.51 (0.28–0.93)	0.028	0.037	0.055	0.78 (0.62–0.98)	0.036
LOC646762	rs117623757	chr7: 29690302	A	0.011	0.030	0.36 (0.15–0.90)	0.028	0.020	0.026	0.67 (0.48–0.93)	0.017
HLA-DQA1	rs7143	chr6: 32610887	T	0.163	0.103	1.33 (1.03–1.72)	0.030	0.083	0.073	1.28 (1.05–1.57)	0.016
HLA-DQA1	rs28538060	chr6: 32611099	T	0.163	0.103	1.33 (1.03–1.73)	0.030	0.083	0.075	1.24 (1.02–1.52)	0.036
HIST1H2BD	rs1059490	chr6: 26171250	C	0.223	0.178	1.38 (1.03–1.84)	0.031	0.207	0.194	1.17 (1.03–1.32)	0.019
COPS6	rs3823641	chr7: 99688220	C	0.037	0.018	2.33 (1.08–5.04)	0.032	0.033	0.031	1.44 (1.08–1.93)	0.014
PLA2G1B	rs5634	chr12: 120762837	G	0.113	0.080	1.57 (1.04–2.38)	0.033	0.102	0.099	1.24 (1.04–1.47)	0.015
CD93	rs2749812	chr20: 23062927	A	0.032	0.060	0.52 (0.28–0.95)	0.033	0.039	0.047	0.70 (0.55–0.89)	0.004
TMEM9	rs147736242	chr1: 201123019	T	0.048	0.073	0.58 (0.35–0.96)	0.035	0.040	0.045	0.77 (0.60–0.99)	0.040
SPEG	rs116911250	chr2: 220337041	A	0.033	0.010	2.52 (1.07–5.93)	0.035	0.083	0.067	1.23 (1.01–1.49)	0.043
SDAD1	rs2242470	chr4: 76878688	A	0.362	0.422	0.78 (0.62–0.99)	0.038	0.374	0.399	0.86 (0.77–0.95)	0.004
METTL14	rs62328061	chr4: 119610606	G	0.167	0.195	0.72 (0.53–0.99)	0.042	0.167	0.168	0.85 (0.74–0.97)	0.016
NRD1	rs1770791	chr1: 52264064	G	0.245	0.198	1.35 (1.01–1.79)	0.043	0.204	0.189	1.16 (1.02–1.32)	0.025
PI4K2B	rs11731839	chr4: 25280727	C	0.208	0.158	1.37 (1.01–1.85)	0.043	0.191	0.172	1.18 (1.03–1.35)	0.016
NAAA	rs4859571	chr4: 76857309	T	0.353	0.412	0.78 (0.61–0.99)	0.043	0.356	0.378	0.87 (0.78–0.96)	0.008
KRT6B	rs388626	chr12: 52841765	G	0.310	0.363	0.78 (0.62–0.99)	0.045	0.281	0.327	0.86 (0.77–0.96)	0.006
PRAM1	rs968501	chr19: 8567500	A	0.207	0.150	1.37 (1.01–1.88)	0.046	0.184	0.180	1.15 (1.00–1.31)	0.043
CPLX4	rs74775550	chr18: 56963479	T	0.263	0.225	1.34 (1.00–1.78)	0.047	0.268	0.236	1.21 (1.08–1.36)	0.001
MICAL1	rs9320288	chr6: 109767930	T	0.440	0.490	0.79 (0.63–1.00)	0.048	0.450	0.480	0.87 (0.77–1.00)	0.046

Notes: OR and *P* value were adjusted for age, sex, and first five principal components. Abbreviations: *CHBVI* = chronic hepatitis B virus infection; *CI* = confidence interval; *OR* = odds ratio.

**Figure 2:**
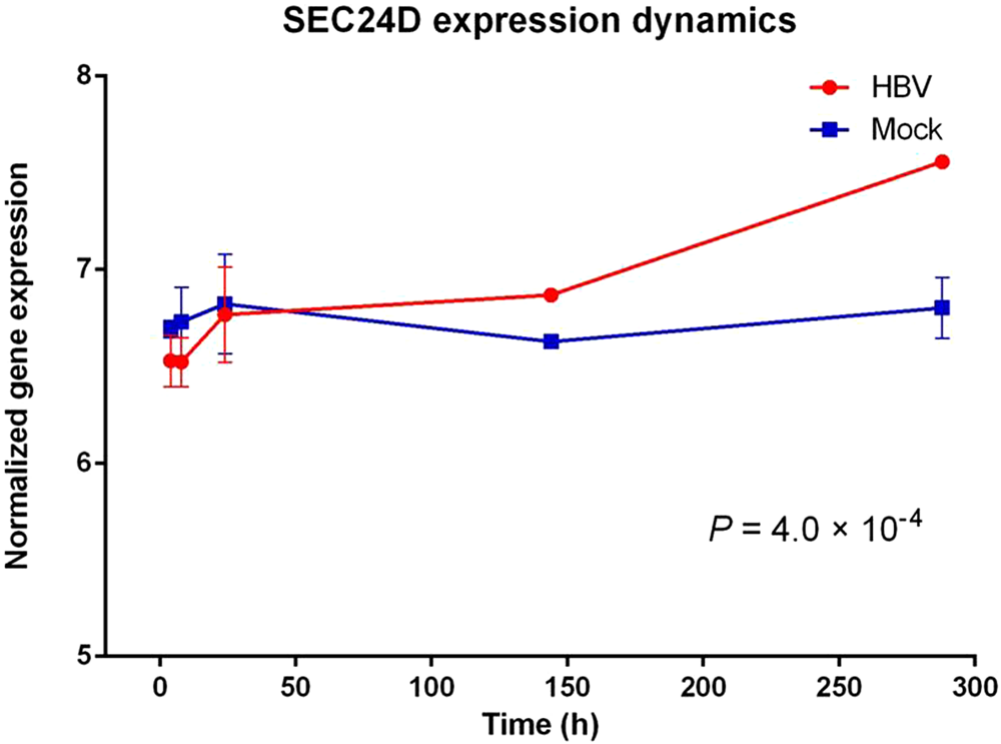
Time course analysis after HBV infection. Gene expression data (log_2_ transformed) were extracted from GEO dataset (Accession Number GSE72068). The mean extent of gene expression for each time point was plotted separately for the HBV and mock-infection groups. Red line represents the HBV-infected group, and blue one represents the mock group. Error bar represents standard deviation (SD).

To identify which genes are more likely to affect HBV infection, we performed time course analysis based on different time points of the expression data from HBV-infected PHHs. We found that only three genes showed significant time-dependent changes in expression in response to HBV (Fig. 2 and Supplementary Fig. 3). The expression of *SEC24D* was cumulative and generally elevated, dependent on the time after HBV infection (*P* = 4 × 10^−4^). At day 12, it had the largest change (>1.5-fold), indicating a potential correlation between *SEC24D* and HBV infection. The extent of expression of *MICAL1* (microtubule associated monooxygenase, calponin and LIM domain containing 1) and *SDAD1* also displayed significant dynamic changes (*P* = 4 × 10^−4^ and *P* = 4 × 10^−2^, respectively). However, *MICAL1* expression was increased within 24 h and decreased after that, indicating different roles in early (24 h and before) and late (post-24 h) responses to HBV infection. For *SDAD1*, the expression changes also displayed a trend of ascending at first and descending latter, again suggesting different responses to HBV infection at different time points and some type of adaptation at day 6. Considering the potentially complex roles of *MICAL1* and *SDAD1* in HBV infection and the main objective of this report, we confined our attention to *SEC24D*.

We found a significant association of *SEC24D* polymorphism with HBV infection (Table 2). In the family sample, when comparing CHBVI with the corresponding sib-control group, SNP rs76459466 (G > T) was negatively associated with HBV infection risk (odds ratio [OR] = 0.64; 95% confidence interval [CI] 0.46, 0.88; *P* = 5.9 × 10^−3^). This association was replicated in an independent case-control sample, which showed that rs76459466 was associated with a significantly lower HBV infection risk (OR = 0.86; 95% CI 0.75, 0.99; *P* = 3.4 × 10^−2^). As there was no significant heterogeneity between the two samples (*P* value of *Q* test > 0.1), meta-analysis was performed on the results from both samples together. We found that the rs76459466 T carriers had a lower risk of HBV infection than the non-carriers (OR_meta_ = 0.82; 95% CI = 0.72, 0.93; *P*_meta_ = 2.0 × 10^−3^). Thus, both genetic association studies and gene expression analyses robustly indicated a potential role of *SEC24D* in HBV infection.

### *SEC24D* inhibits HBV replication

*SEC24D* is a member of the SEC24 subfamily and correlates with vesicle trafficking. According to the RNA-Seq Atlas database^29^, *SEC24D* is expressed in various tissues, including the liver. However, the specific role of *SEC24D* in HBV infection has not been illuminated. Therefore, we investigated its impact on HBV infection using *in vitro* functional experiments.

**Figure 3:**
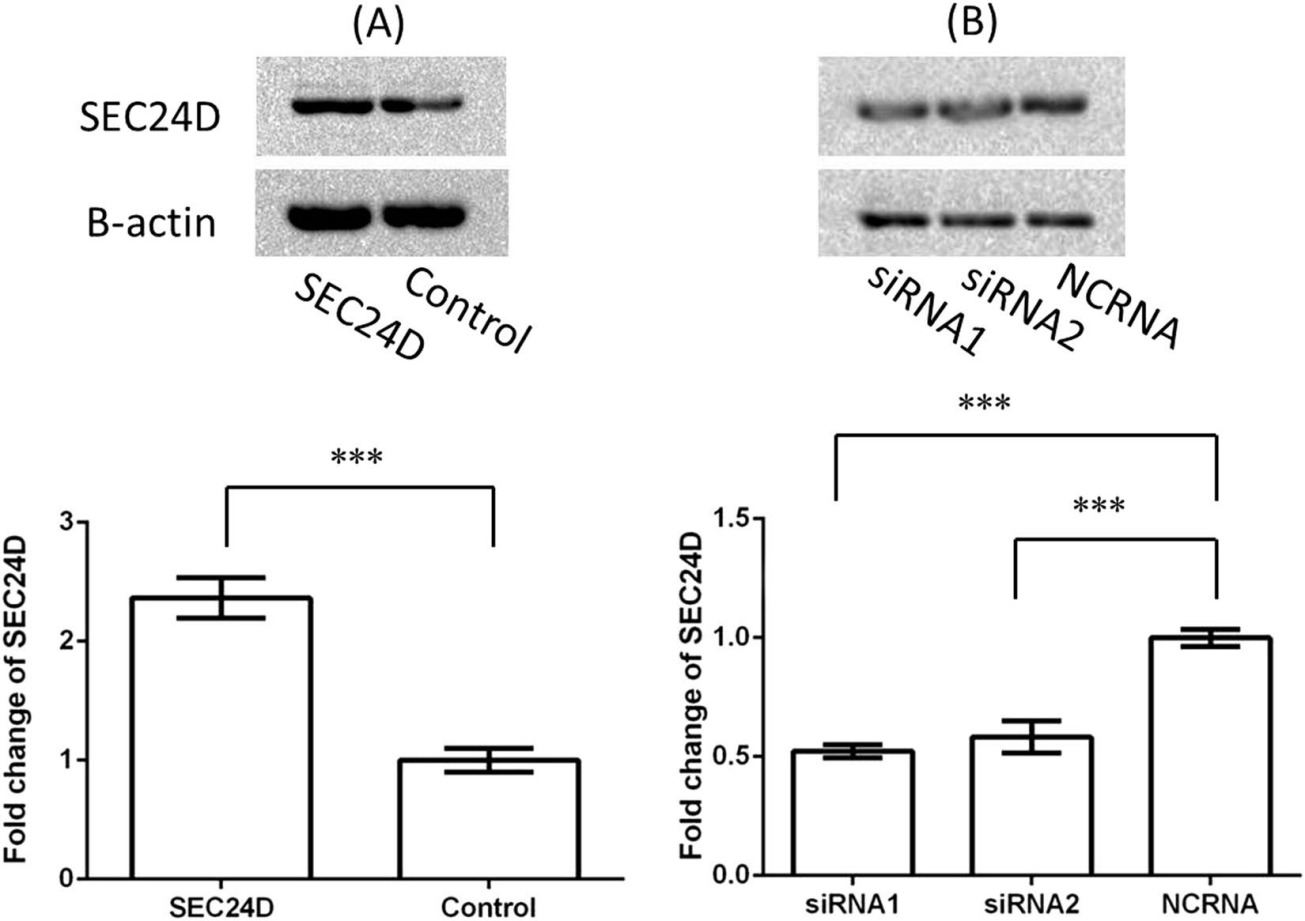
Western blotting analysis for amount of *SEC24D* protein in HepG2 cells. Cells (~2 × 10^5^) were transfected by pGEM-4Z-HBV1.3, together with pEGFP-C3-*SEC24D* (*SEC24D*) or pEGFP-C3 control vectors (Control) (**A**) or with *SEC24D*-specific siRNAs (siRNA1 and siRNA2) or negative control siRNAs (NCRNAs) (**B**). Cell lysates were collected after 48 h transfection. Error bar represents SD; *P < 0.05, **P < 0.01, and ***P < 0.001.

**Figure 4:**
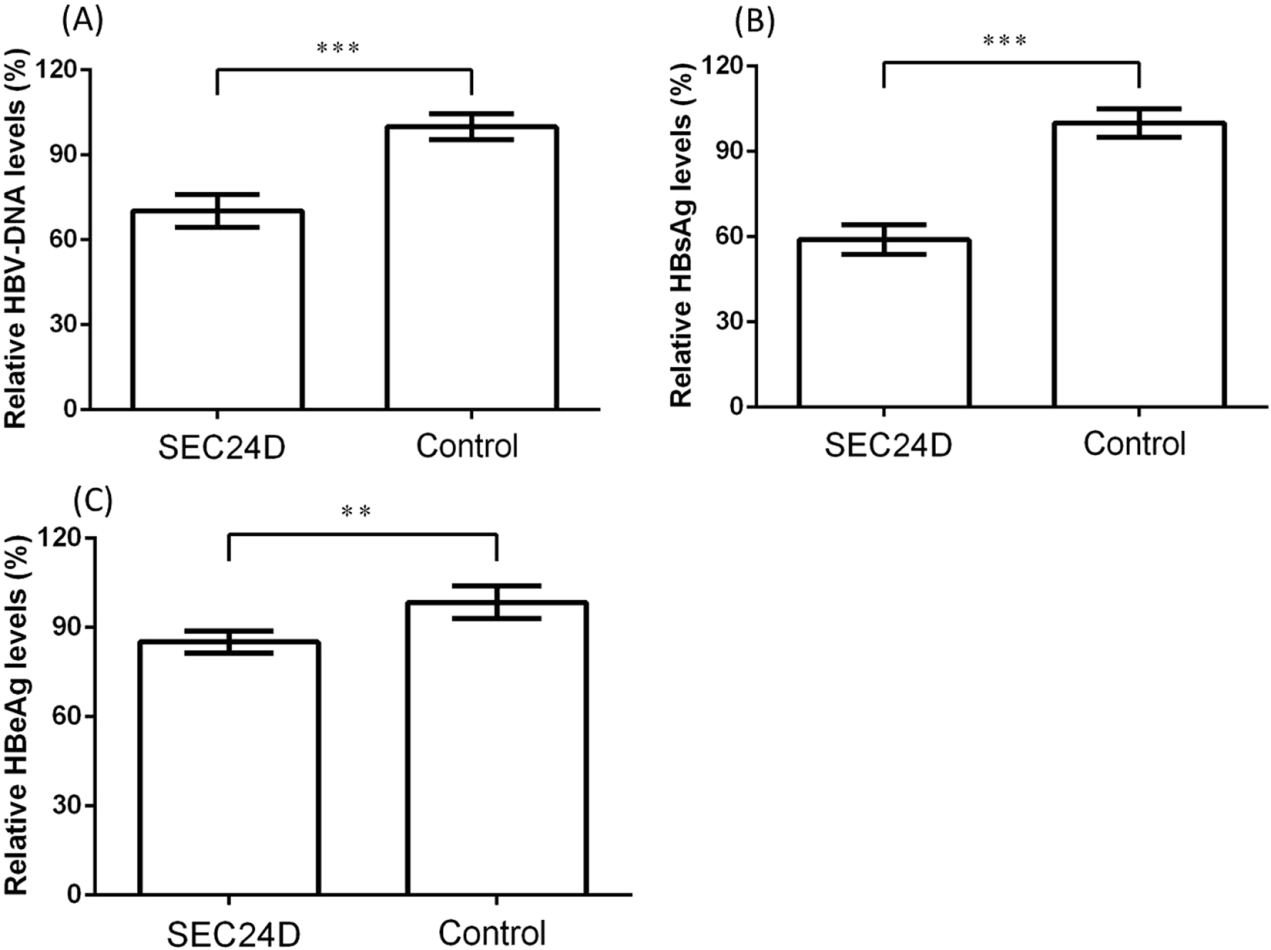
Overexpressed *SEC24D* inhibited HBV replication in HepG2 cells. Amount of HBV-DNA was detected by quantitative real-time PCR (**A**), and the quantities of HBsAg (**B**) and HBeAg (**C**) were tested by chemiluminescent microparticle immunoassay. All the supernatant liquids were collected after 48 h of transfection. Error bar represents SD; *P < 0.05, **P < 0.01, and ***P < 0.001.

**Figure 5:**
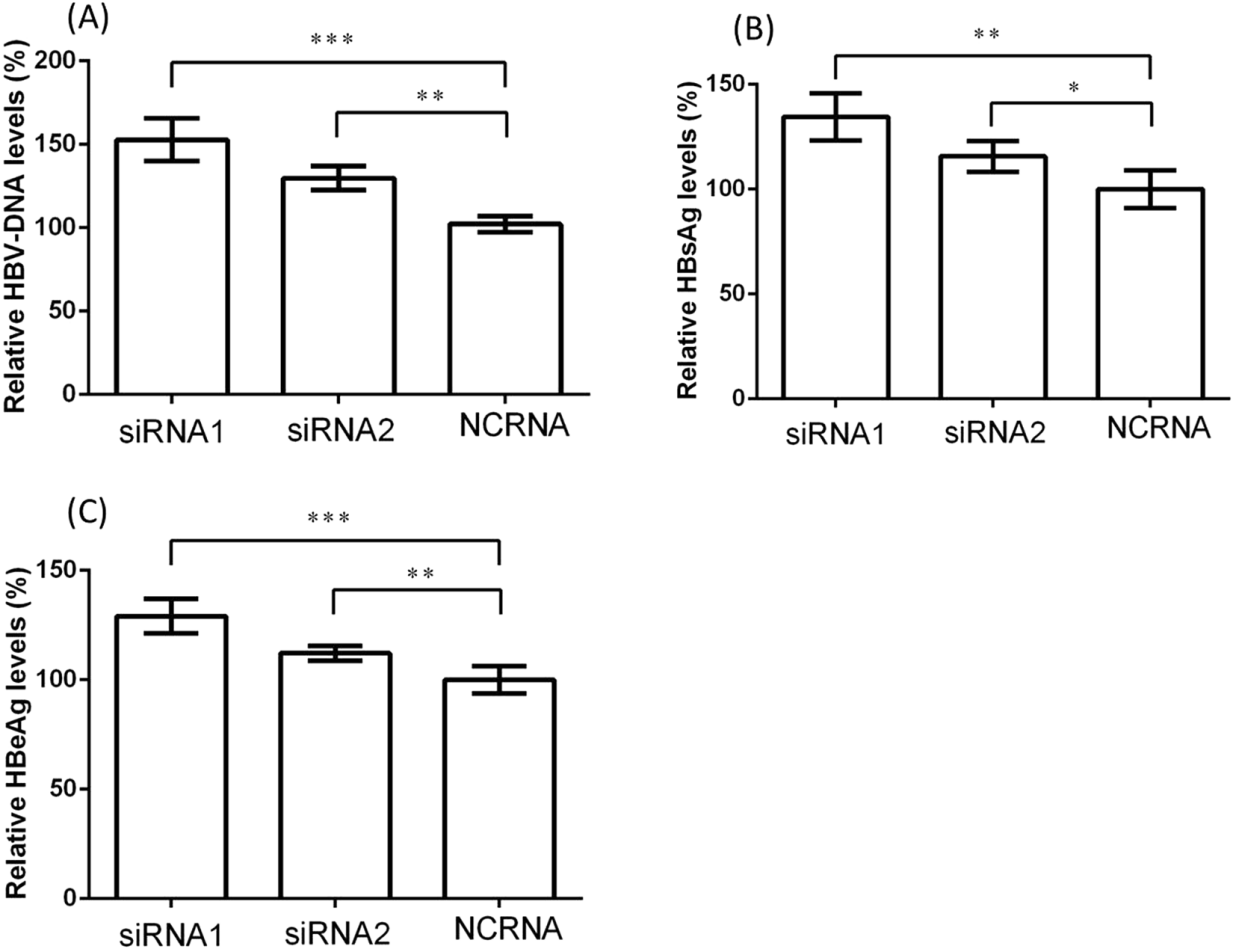
Inhibition of *SEC24D*-enhanced HBV replication in HepG2 cells. *SEC24D* expression was inhibited by two independent siRNAs. The amount of HBV-DNA was detected by quantitative real-time PCR (**A**), and the amounts of HBsAg (**B**) and HBeAg (**C**) were measured by chemiluminescent microparticle immunoassay. All the supernatant liquids were collected after 48 h of transfection. Error bar represents SD; *P < 0.05, **P < 0.01, and ***P < 0.001.

To explore the potential effect of *SEC24D* on HBV infection, we investigated the amounts of HBV-DNA, HBsAg, and HBeAg in the cell medium after *SEC24D* overexpression or inhibition. The HepG2 cells were transfected by pGEM-4Z-HBV1.3, together with either pEGFP-C3-*SEC24D* or pEGFP-C3 control plasmids (Figs 3A and 4A–C). Compared with the cells treated with control vectors, the cells with overexpressed *SEC24D* plasmid showed a significant drop in HBV-DNA load to 70.3 ± 5.8%, HBsAg to 59.0 ± 5.1%, and HBeAg to 85.1 ± 3.7%. For the *SEC24D* inhibition (Figs 3B and 5A–C), we used two independent siRNAs, which led to markedly enhanced amounts of HBV-DNA to 152.8 ± 12.9% and 129.8 ± 7.0%, HBsAg to 134.6 ± 11.2% and 115.7 ± 7.4%, and HBeAg to 129.1 ± 7.9% and 112.2 ± 3.3%. Further, we replicated the antiviral effect of *SEC24D* efficiently resisting HBV in HepG2.2.15 cells (Supplementary Figs 4–6). The HBV markers were significantly reduced by *SEC24D* overexpression but increased by *SEC24D* inhibition.

### Decreased *SEC24D* expression in infected liver tissues

**Figure 6:**
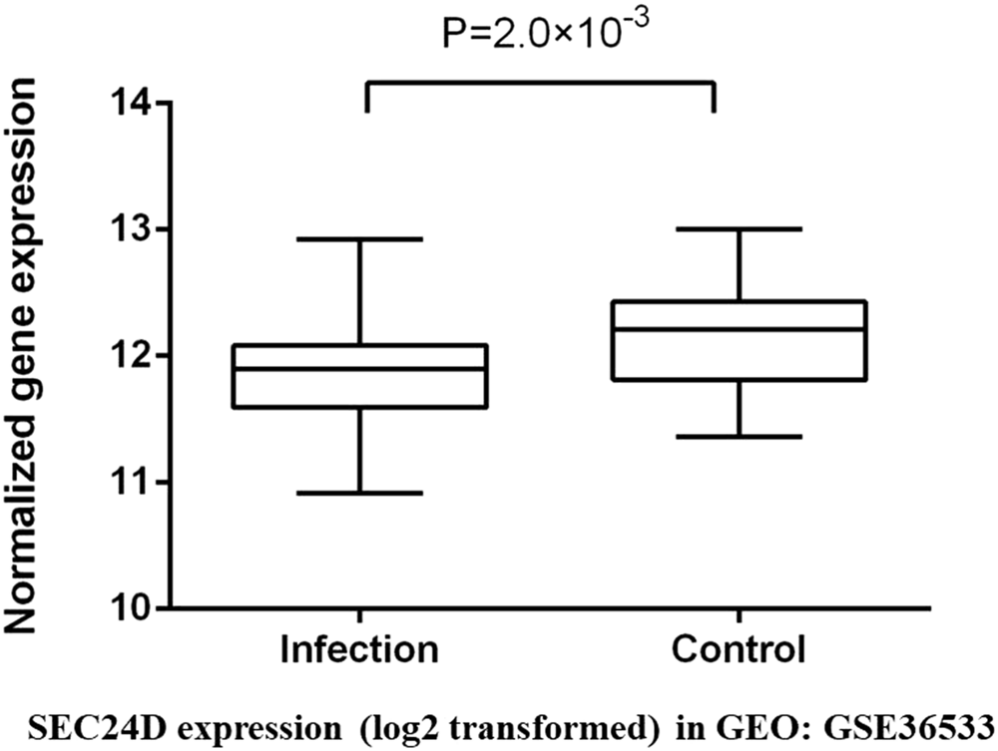
Expression of *SEC24D* in liver tissues of infected and control groups (Accession Number GSE36533). Top bar is maximum expression, lower bar is minimum observation, top of box is third quartile, bottom of box is first quartile, middle bar is median value.

To further confirm the role of *SEC24D* in HBV infection, we examined whether a differential degree of *SEC24D* expression existed in liver tissues from woodchucks by searching public database. The woodchuck can be naturally infected with woodchuck hepatitis virus (WHV), a hepadnavirus that is genetically close to human HBV. It is often used as an animal model for studying the pathogenesis of CHBVI and HBV-related HCC development in human^30^. We investigated *SEC24D* expression in the infected (n = 60) and non-infected (n = 63) liver tissues of WHV models (Fig. 6). When compared with the control group (mean log_2_ normalized expression value of 11.90), we found obviously lower *SEC24D* expression (mean log_2_ normalized expression value of 12.15) in the infected liver (fold change 1.2; *P* = 0.002). Consistent with our previous findings, these data support the protective role of *SEC24D* in HBV infection.

## Discussion

During the past several years, GWAS has been commonly used to investigate the genetic predisposition to common diseases, but the identified susceptibility variants can explain only a small proportion of the known heritability. Using new research strategies would be helpful to identify the real causal variants. In this study, we combined WES data, iSelect-based array data, and GEO expression profiles followed by *in vitro* experiments to identify novel susceptibility genes.

In the discovery stage, we used sTDT analysis of WES data to identify SNPs that were significant in 300 CHBVIs compared with 300 unaffected siblings. The primary reason for choosing sib-pairs as the discovery sample for exome-sequencing analysis was the fact that the family-based design has an advantage over the case-control design for its robustness to population stratification^31^. Then the top 4000 SNPs with the smallest *P* values were selected for subsequent replication. In the replication stage, these SNPs were genotyped in an independent sample consisting of 1648 CHBVIs and 1439 unrelated controls. We found that 36 nominal SNPs located in 31 genes were validated (*P* < 0.05) with the same association directions observed in the discovery stage. To narrow down the candidates for the causative genes, we performed time course analysis to investigate the expression of genes that displayed significant time-related changes induced by HBV. We found that, as time went on, only *SEC24D* expression was markedly increased after HBV infection. Moreover, based on the aforementioned genetic association study, SNP rs76459466 in *SEC24D* was significantly associated with HBV infection in both family and case-control samples, which also supports *SEC24D* as a susceptibility gene for HBV infection. Taken together, the association study and the time course analysis indicated that *SEC24D* is a potential candidate gene for encouraging HBV infection.

*SEC24D* is located on chromosome 4q26 and encodes the protein involved in vesicle trafficking that is supposed to affect the HBV infection process^32^. *SEC24D* inhibition is involved in enteropathogenic *Escherichia coli*- and enterohemorrhagic *E*. *coli*-induced diseases^33^. However, there is no report regarding the association between *SEC24D* and HBV infection. By overexpressing or inhibiting *SEC24D* expression, we examined whether the amounts of HBV markers changed. By enhancing *SEC24D* expression, we found that the protein significantly reduced the amounts of HBV-DNA, HBsAg, and HBeAg. Consistently, inhibition of *SEC24D* by two independent siRNAs produced significantly greater amounts of HBV-DNA, HBsAg, and HBeAg. These data indicate that *SEC24D* plays a protective role against HBV infection. Furthermore, the expression profiles of WHV models in liver tissue showed decreased expression in infected tissues compared with non-infected tissues, which further supports the antiviral role of *SEC24D* in HBV-exposed persons. Considering the important role of *SEC24D* in HBV infection, we then explored the possible biological pathway involved by analyzing expression data of liver tissues from 96 patients with HBV-related HCC (Supplementary Tables 1 and 2). When comparing samples with high *SEC24D* expression with those with low expression, we found 262 differential genes (false discovery rate [FDR] *Q* value < 0.005, |fold change| > 1.5) that were significantly changed after *SEC24D* dysregulation. Interestingly, the following pathway enrichment analysis showed the most significant pathway to be fatty acid degradation. It has been reported that fatty acid biosynthesis is involved in replication of the hepatitis C virus genome, as well as HBV proliferation^34^. Moreover, saturated fatty acids could inhibit HBV replication mediated by the innate immune response via Toll-like receptor 4^35^. Thus, we speculate that *SEC24D* inhibits HBV replication through increasing the amount of saturated fatty acid. However, such an antiviral signaling pathway needs to be verified.

Although we identified *SEC24D* as a novel gene for HBV infection, some of limitations in this study need to be considered. First, we restricted our search to the candidate genes with constantly enhanced expression after HBV infection. However, some genes may be involved only in the early response to HBV, and their expression would not keep increasing at all time points. Thus, more comprehensive studies are needed to uncover new genes. Second, although we selected the top 4000 SNPs for replication from WES analysis of family samples, only 36 SNPs showed nominal association with HBV infection. Such a relatively low replication might be attributable to differences in both techniques (sequencing and array) and samples (family vs. case-control). Third, although we found that SNP rs76459466 in *SEC24D* played a protective role in HBV infection, we did not investigate the potential biological functions of this SNP. To provide clearer pathogenic insights into HBV infection, the biological functions of this and other SNPs in this gene merit further investigation. In spite of these potential limitations, we integrated genetic association studies, expression data, and *in vitro* functional assays to minimize false-positive association^36^.

In conclusion, we first revealed *SEC24D* as a novel gene crucial for HBV infection by employing an integrated functional genomics strategy. Future studies, such as pathway analysis and SNP functional study, are needed to better define the mechanisms of this gene’s actions in HBV infection.

### Declarations

Ethics approval and consent to participate: This project was approved by the Ethical Committee of the First Affiliated Hospital of Zhejiang University School of Medicine. Informed written consent was obtained from every participant.

## Supplementary information

Supplementary-Figures

Supplementary-Tables

Supplement-Code and parameter setting for bioinformatics analysis

Supplement-Genotype-datasets

## Data Availability

We downloaded bioinformatics tools and data from public domains with the details given in the Methods section. All materials generated from this project are available upon the request. The WES data has been submitted to NCBI databank with accession code PRJNA553618. The genotyping data is provided in Supplementary Dataset.
