# Supplementary material for: Identification and characterization of SEC24D as a susceptibility gene for hepatitis B virus infection
Source: Sci Rep. 2019 Sep 17;9:13425. doi: 10.1038/s41598-019-49777-8 (PMC6748997; doi:10.1038/s41598-019-49777-8)
Supplement: Supplementary file 1 — Supplementary-Figures [file 41598_2019_49777_MOESM1_ESM.pdf]

Identification and characterization of *SEC24D* as a susceptibility gene for hepatitis B virus infection

Xianzhong Jiang, Bin Zhang, Junsheng Zhao, Yi Xu, Haijun Han, Kunkai Su, Jingjing Tao, Rongli Fan, Xinyi Zhao, Lanjuan Li, and Ming D. Li

Legends to Supplementary Figures:

Supplementary Figure 1. The principal components analysis (PCA) of family and population samples. (A) PCA in 300 sib-pairs. (B) PCA in 3087 population samples. Notes: Principal component (PC) 1 versus PC2 for each sample set. Red cross represents the chronic hepatitis B virus infection (CHBVI) cases, and blue one represents the control group.

Supplementary Figure 2. Sequencing depth of the 300 sib-pairs.

Supplementary Figure 3. Time course analysis after HBV infection. Notes: Gene expression data (log2 transformed) was extracted from GEO dataset (GSE72068). The mean gene expression levels for each time point were plotted separately for the mock and HBV groups. Red line represents the HBV-infected group, and blue one represents the mock group. Error bar represents s.d..

Supplementary Figure 4. Western blotting analysis for the protein levels of SEC24D in HepG2.2.15 cells. Notes: Cells (~2×105) were transfected by pGEM-4Z-HBV1.3, together with pEGFP-C3-SEC24D (SEC24D) or pEGFP-C3 control vectors (Control) (A), or with SEC24D-specific siRNAs (siRNA1 and siRNA2) or negative control siRNAs (NCRNAs) (B). The cell lysates were collected after 48 h transfection. Error bars represents s.d.. *P < 0.05, **P < 0.01, and ***P < 0.001.

Supplementary Figure 5. Overexpressed SEC24D inhibited HBV replication in HepG2.2.15 cells. Notes: The levels of HBV-DNA were detected by quantitative real-time PCR (A), the HBsAg (B) and HBeAg (C) were tested by chemiluminescent microparticle immunoassay. All the supernatants were collected after 48 h transfection. Error bars represents s.d.. *P < 0.05, **P < 0.01, and ***P < 0.001.

Supplementary Figure 6. Inhibition of SEC24D enhanced HBV replication in HepG2.2.15 cells. Notes: SEC24D expression was inhibited by two independent siRNAs. The levels of HBV-DNA were detected by quantitative real-time PCR (A), the HBsAg (B) and HBeAg (C) were tested by chemiluminescent microparticle immunoassay. All the supernatants were collected after 48 h transfection. Error bars represents s.d.. *P < 0.05, **P < 0.01, and ***P < 0.001.

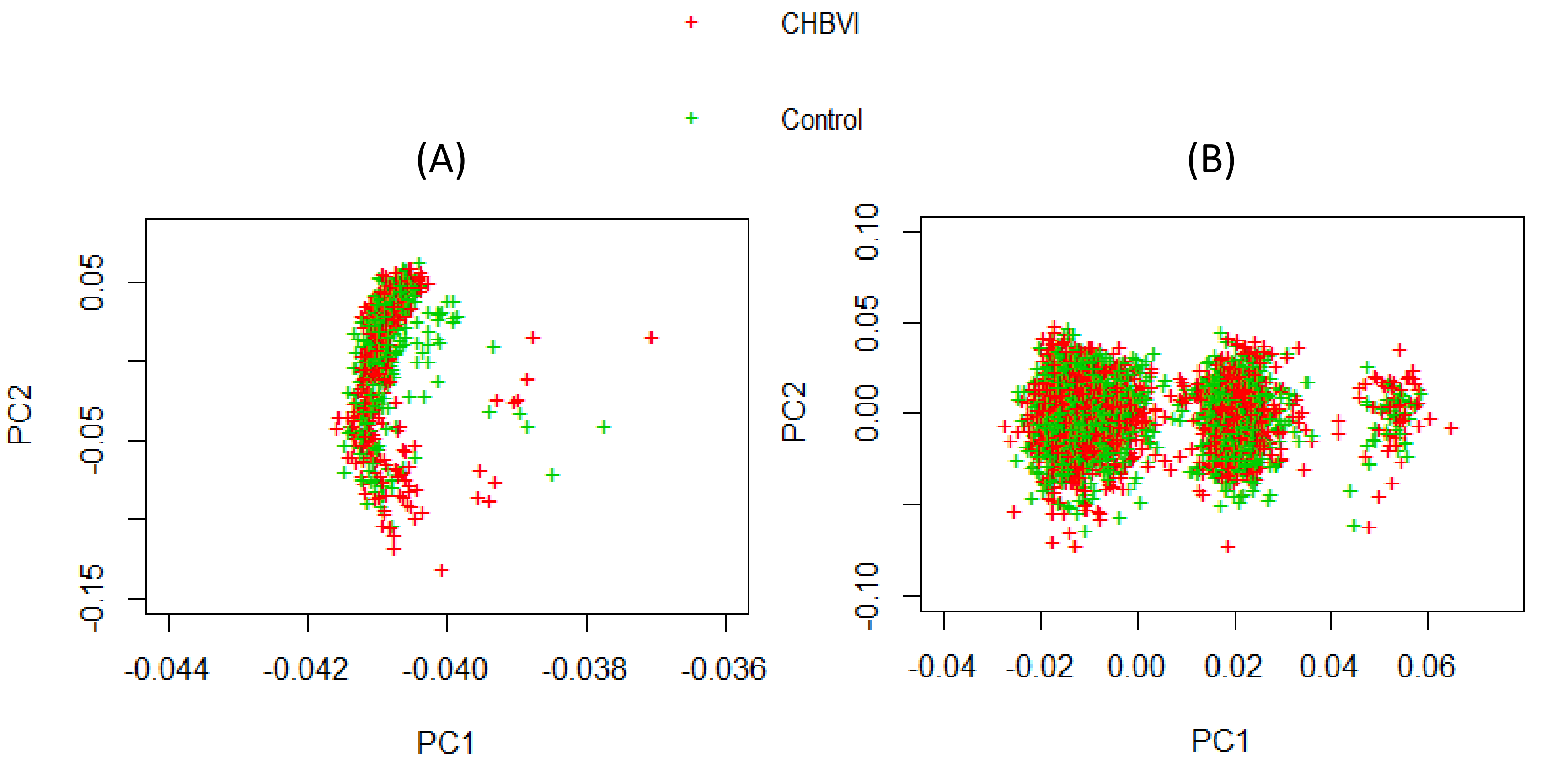

Supplementary Figure 1

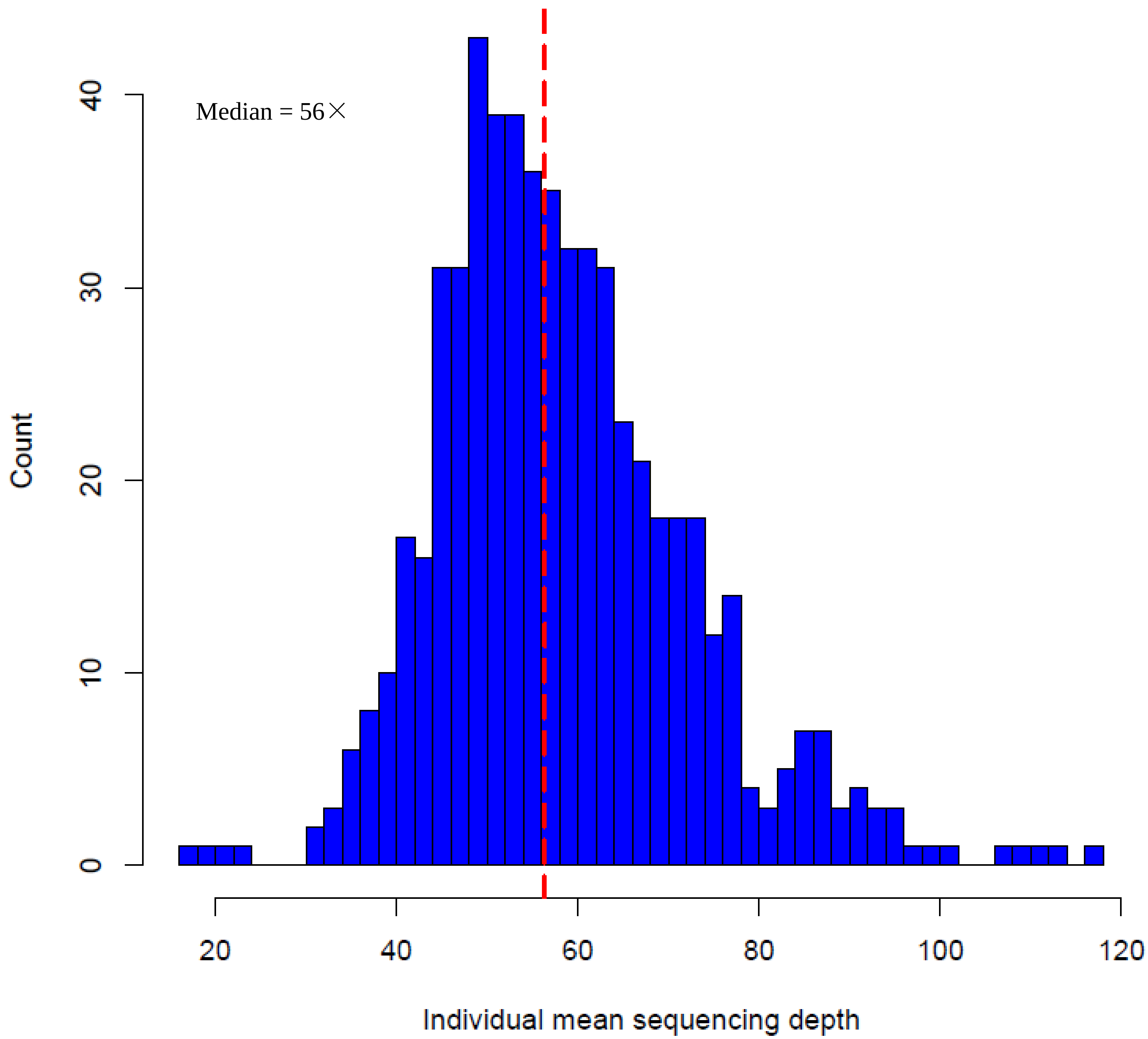

Supplementary Figure 2

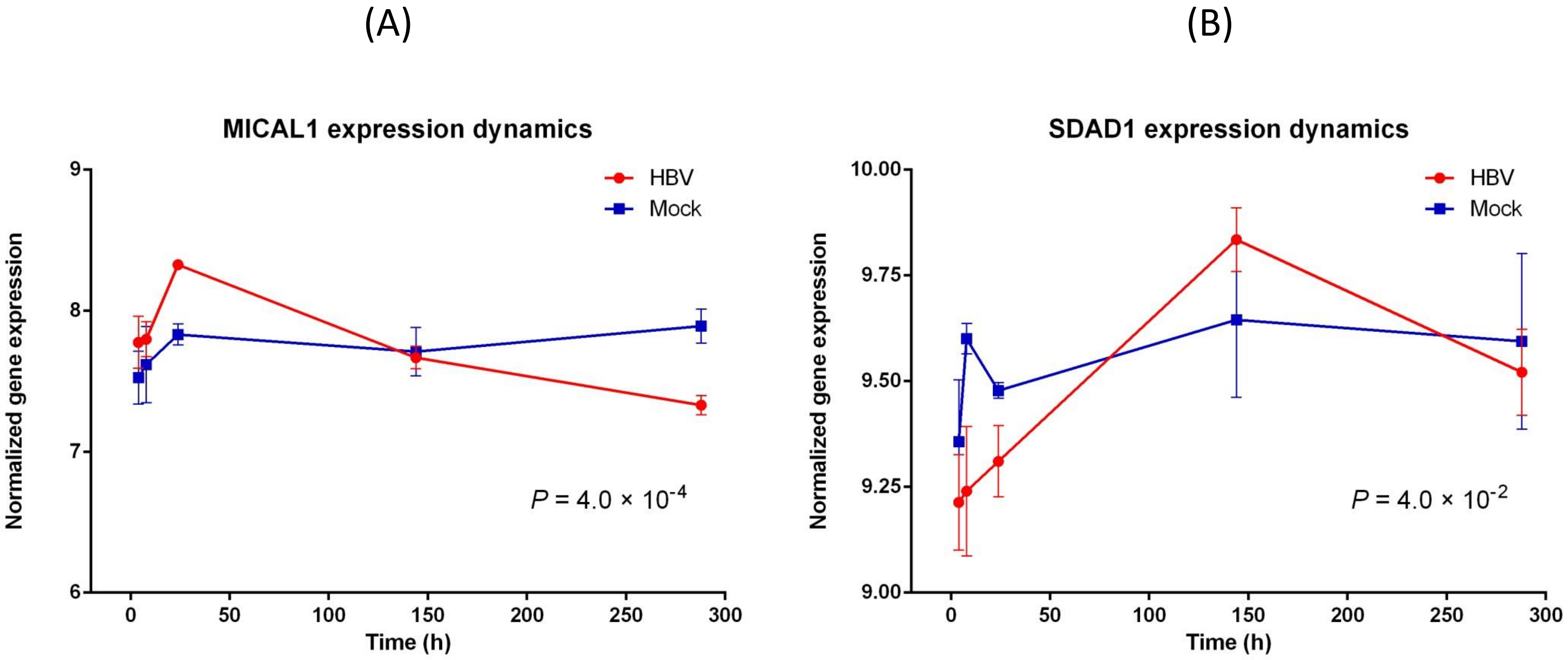

Supplementary Figure 3

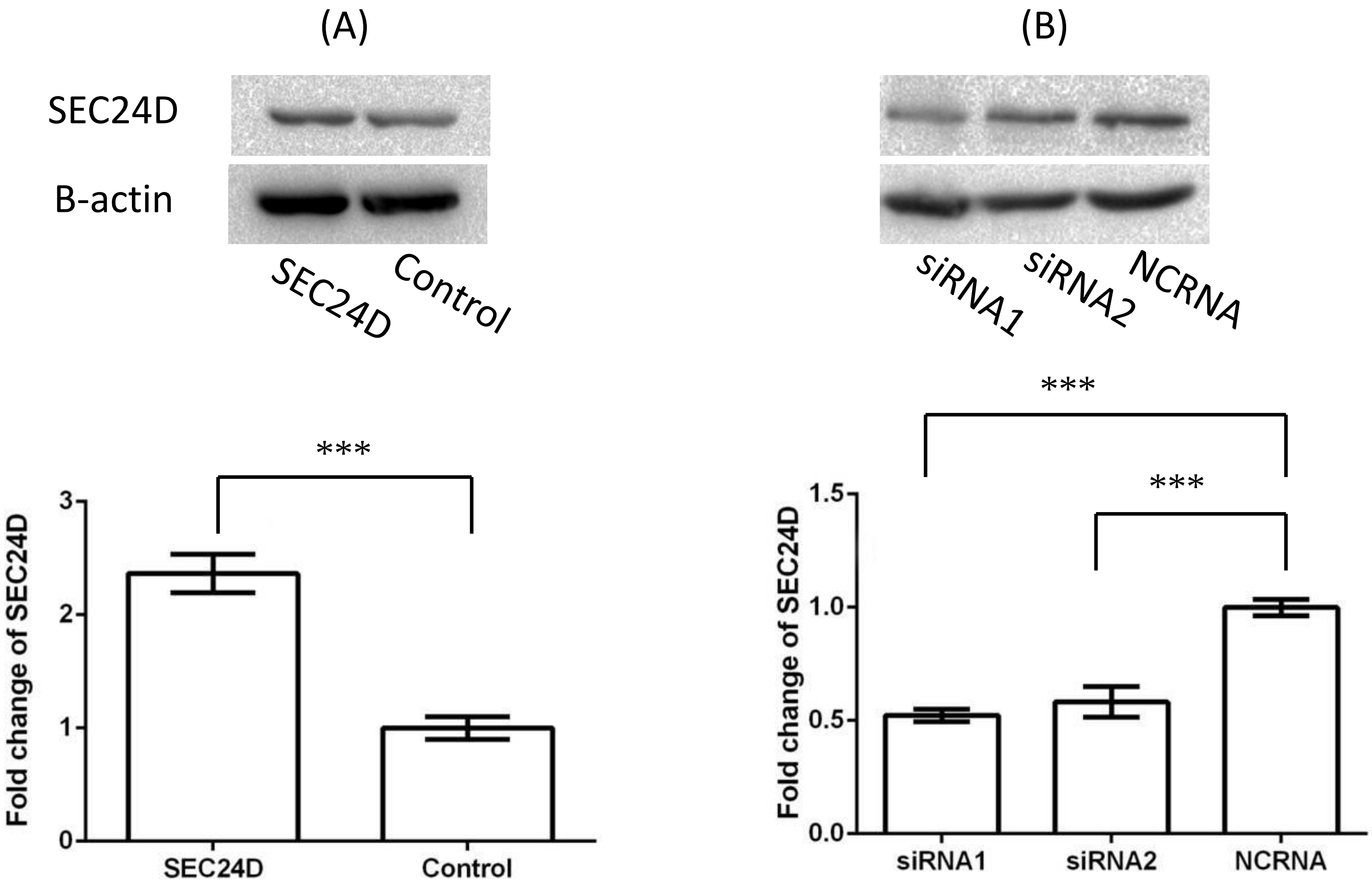

Supplementary Figure 4

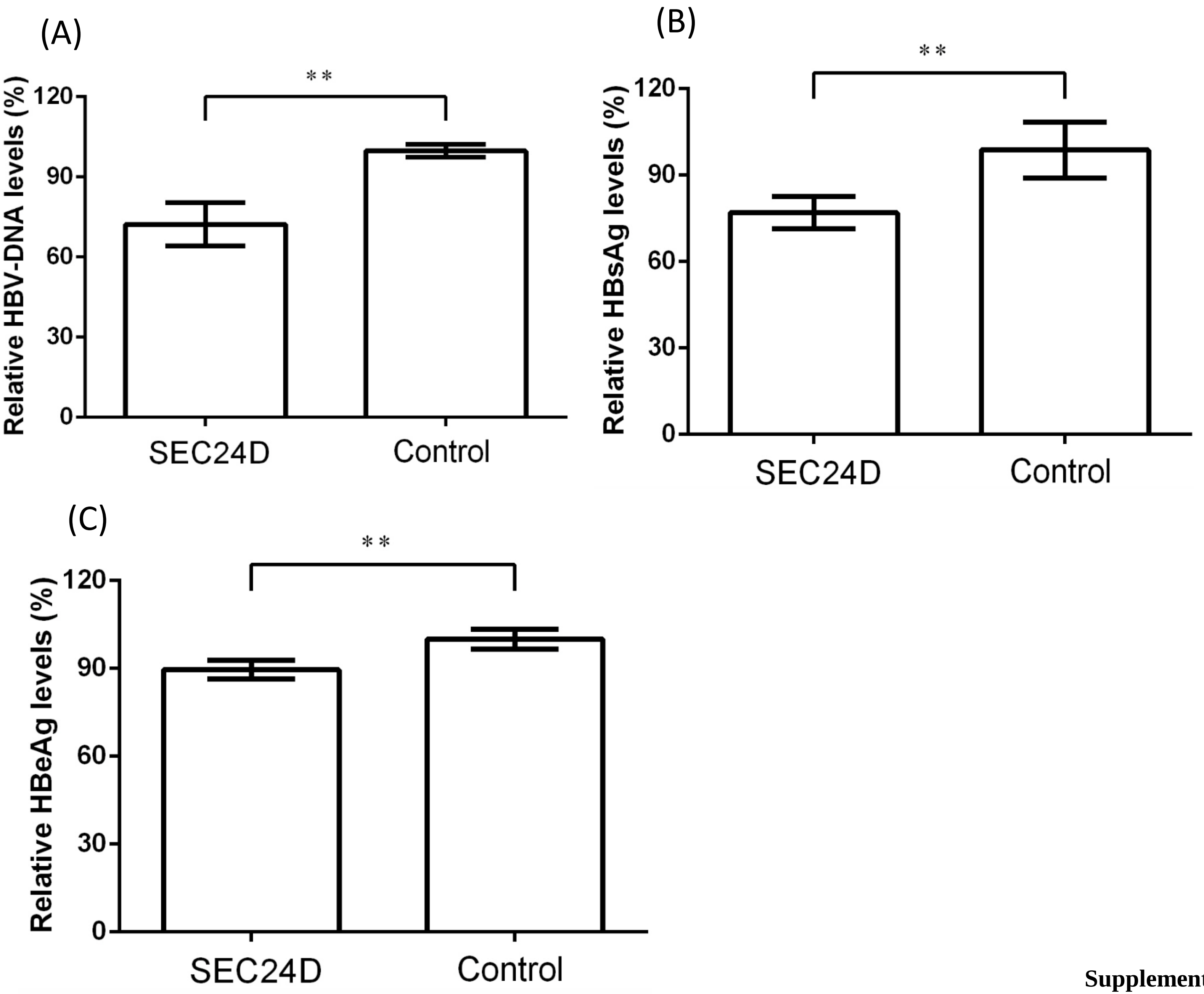

Supplementary Figure 5

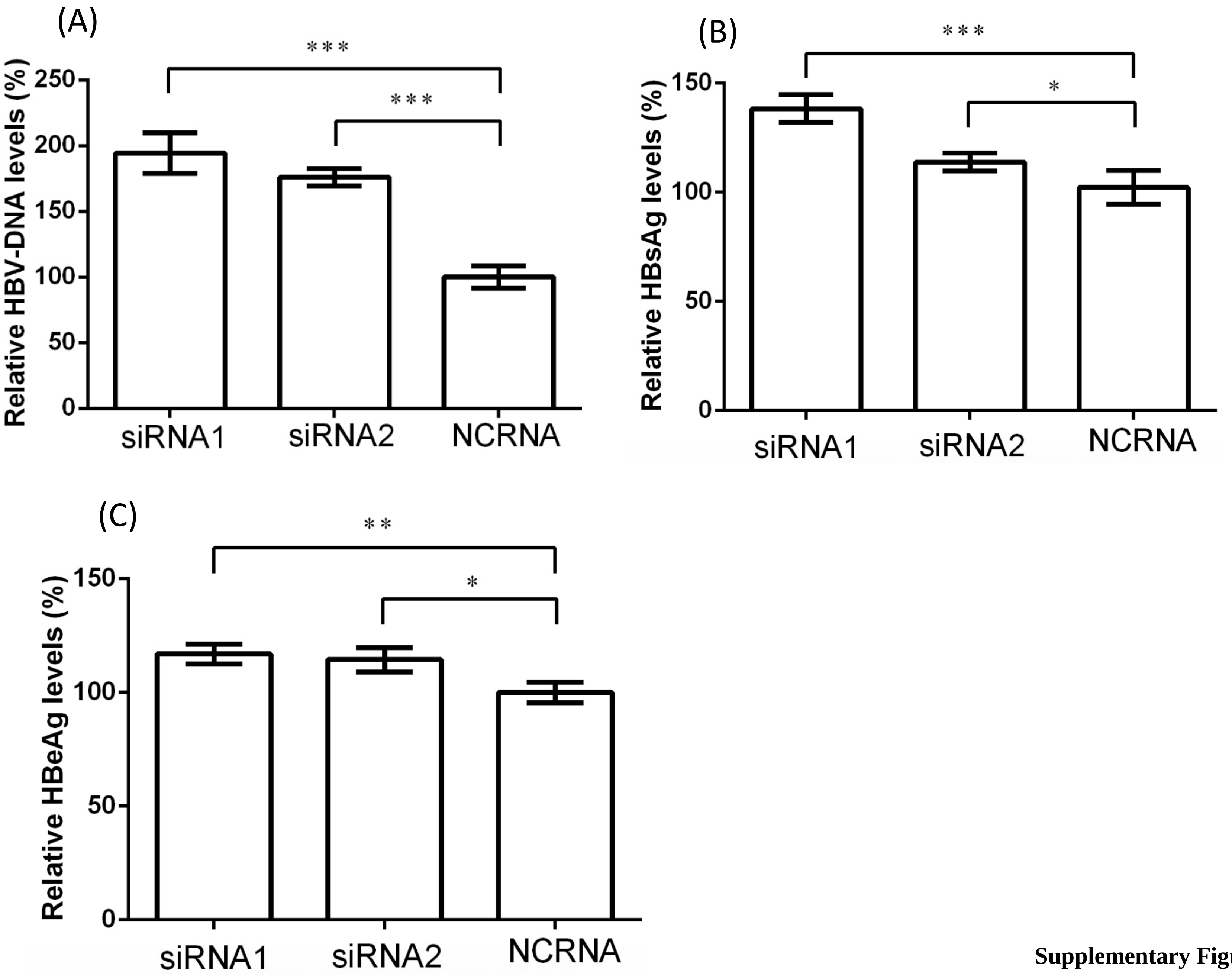

Supplementary Figure 6
