# Supplementary material for: Identification and characterization of SEC24D as a susceptibility gene for hepatitis B virus infection
Source: Sci Rep. 2019 Sep 17;9:13425. doi: 10.1038/s41598-019-49777-8 (PMC6748997; doi:10.1038/s41598-019-49777-8)
Supplement: Supplementary file 3 — Supplement-Code and parameter setting for bioinformatics analysis [file 41598_2019_49777_MOESM3_ESM.pdf]

Identification and characterization of *SEC24D* as a susceptibility gene for hepatitis B virus infection

Xianzhong Jiang,[1)] Bin Zhang,[1)] Junsheng Zhao,[1)] Yi Xu,[1)] Haijun Han,[1)] Kunkai Su,[1)] Jingjing Tao,[1)] Rongli Fan,[1)] Xinyi Zhao,[1)] Lanjuan Li,[1)] and Ming D. Li[1,2)]

1) State Key Laboratory for Diagnosis and Treatment of Infectious Diseases, Collaborative Innovation Center for Diagnosis and Treatment of Infectious Diseases, The First Affiliated Hospital, Zhejiang University School of Medicine, Hangzhou, China; and 2) Research Center for Air Pollution and Health, Zhejiang University.

Keywords: Functional genomics, hepatitis B virus, *SEC24D*, single nucleotide polymorphism

Running title: *SEC24D* and susceptibility to HBV infection

Emails: Xianzhong Jiang: jiangxianzhong@126.com; Bin Zhang: 21718148@zju.edu.cn; Junsheng Zhao: 11818155@zju.edu.cn; Yi Xu: xuyi714@126.com; Haijun Han: haijunhan_happy@126.com; Kunkai Su: kunkai_su@hotmail.com; Jingjing Tao: taojingjing851016@163.com; Rongli Fan: fanrongli2013@163.com; Xinyi Zhao: xinyizhao@zju.edu.cn; Lanjuan Li: ljli@zju.edu.cn; Ming D. Li: ml2km@zju.edu.cn

Corresponding author:

1) Professor Ming D. Li, PhD, State Key Laboratory for Diagnosis and Treatment of Infectious Diseases, The First Affiliated Hospital, Zhejiang University School of Medicine, Hangzhou, China. E-mail: ml2km@zju.edu.cn

2) Professor Lanjuan Li, M.D. State Key Laboratory for Diagnosis and Treatment of Infectious Diseases, The First Affiliated Hospital, Zhejiang University School of Medicine, Hangzhou, China. Email: ljli@zju.edu.cn

Code and parameter setting for bioinformatics analysis:

In the replication stage, association of SNPs with HBV infection was performed under an additive genetic model using PLINK (v.1.07) with age, sex and the first five PCs as covariates. The input parameters used in current study as following:

```
nohup plink --bfile HBV_infection_file --pheno HBV_phenotype --pheno-name
HBV_infection --geno 0.05 --mind 0.05 --noweb --logistic --covar
covariate_phenotype_file --covar-name age, sex, PC1, PC2, PC3, PC4, PC5
--nonfounders --maf 0.05 --hwe 0.000001 --adjust --out results_HBV_infections &
```

The population admixture of samples was assessed by PC analysis (PCA) as implemented in EIGENSTRAT. The input parameters used in current study as following:

```
nohup smartpca.perl -i HBV_infection_file.bed -a HBV_infection_file.bim -b
HBV_infection_file.fam -o HBV_infection_file.pca -p HBV_infection_file.pca.plot -e
HBV_infection_file.pca.eval -l HBV_infection_file.pca.log -k 10 -s 10 -m 0 &
```
